# Phenotypic and Molecular Characterization of *Brucella microti*-Like Bacteria From a Domestic Marsh Frog (*Pelophylax ridibundus*)

**DOI:** 10.3389/fvets.2018.00283

**Published:** 2018-11-15

**Authors:** Maryne Jaý, Guillaume Girault, Ludivine Perrot, Benoit Taunay, Thomas Vuilmet, Frédérique Rossignol, Pierre-Hugues Pitel, Elodie Picard, Claire Ponsart, Virginie Mick

**Affiliations:** ^1^ANSES/Paris-Est University, EU/OIE/FAO and National Reference Laboratory for Brucellosis, Animal Health Laboratory, Maisons-Alfort, France; ^2^DDPP de la Drôme, Valence, France; ^3^LABEO Caen, France; ^4^LABEO-Orne Alençon, France

**Keywords:** Brucellosis, *Brucella microti*, domestic frog, *Pelophylax ridibundus*, Europe

## Abstract

Several *Brucella* isolates have been described in wild-caught and “exotic” amphibians from various continents and identified as *B. inopinata*-like strains. On the basis of epidemiological investigations conducted in June 2017 in France in a farm producing domestic frogs (*Pelophylax ridibundus*) for human consumption of frog's legs, potentially pathogenic bacteria were isolated from adults showing lesions (joint and subcutaneous abscesses). The bacteria were initially misidentified as *Ochrobactrum anthropi* using a commercial identification system, prior to being identified as *Brucella* spp. by MALDI-TOF assay. Classical phenotypic identification confirmed the *Brucella* genus, but did not make it possible to conclude unequivocally on species determination. Conventional and innovative bacteriological and molecular methods concluded that the investigated strain was very close to *B. microti* species, and not *B. inopinata*-like strains, as expected. The methods included growth kinetic, antimicrobial susceptibility testing, RT-PCR, Bruce-Ladder, Suis-Ladder, RFLP-PCR, AMOS-ERY, MLVA-16, the ectoine system, 16S rRNA and *recA* sequence analyses, the LPS pattern, *in silico* MLST-21, comparative whole-genome analyses (including average nucleotide identity ANI and whole-genome SNP analysis) and HRM-PCR assays. Minor polyphasic discrepancies, especially phage lysis and A-dominant agglutination patterns, as well as, small molecular divergences suggest the investigated strain should be considered a *B. microti-*like strain, raising concerns about its environmental persistence and unknown animal pathogenic and zoonotic potential as for other *B. microti* strains described to date.

## Introduction

Based on bacteriological features, host preference and pathogenicity, the taxonomy of the *Brucella* genus (http://www.bacterio.net/brucella.html) currently identifies 12 species split into (*i*) “core” *Brucella* species, including the six “classical” species (*Brucella melitensis, B. abortus, B. suis, B. canis, B. ovis, B. neotomae*; http://www.oie.int/fr/normes/code-terrestre/acces-en-ligne/ < underline)>, *B. ceti* and *B. pinnipedialis* isolated from marine mammals (1, 2), and the recently described *B. papionis* from baboons (3), and *ii*) the emerging atypical *Brucella* species (4–6). The atypical *Brucella* species include fast-growing *B. microti* initially isolated from common voles (7) and reported from soil (8) and red fox (9), *B. inopinata* BO1 isolated from a breast implant (10), *B. vulpis* from red fox (11), as well as, unclassified isolates: BO2 isolated from a patient with chronic destructive pneumonia (12), probably representing a novel lineage of *B. inopinata*, and novel Australian rodent isolates (13). Interestingly, the atypical *Brucella* isolates are phenotypically close to *Ochrobactrum* spp., a soil-associated facultative human pathogen (14), but genetically close to the *Brucella* genus. Molecular data show that Australian rodent isolates are related to *B. inopinata* and strain BO2, although *B. microti* is genetically close to the core phylogenetic clade of *Brucella*, especially to *B. suis* 1330 (15).

*Brucella* infections have been described in wild-caught and captive-bred anuran species native to Africa, South and Central America, and Australia, from animals showing systemic or localized infections (16–22), as well as, from other apparently healthy individuals (23). These exotic frog strains are affiliated with the atypical *Brucella* group, genetically close to *B. inopinata* (24), (18).

Although human infections due to *B. inopinata* have been reported (10, 12), its zoonotic potential remains unclear. Likewise, the pathogenicity of atypical *Brucella* bacteria and their transmission among amphibians are unknown (25).

This study presents the isolation and phenotypic identification of a new *Brucella* field isolate from *Pelophylax ridibundus*, a domestic frog on a breeding farm, as well as, its in-depth genomic characterization.

## Results and discussion

### Detection of a presumptive brucella field isolate from the domestic frog *p. ridibundus*

In June 2017, epidemiological investigations were conducted for research purposes on a frog farm in France breeding the first domesticated strain of *P. ridibundus* Rivan92®, selected by the French National Institute for Agricultural Research (INRA) for human consumption (frog's legs). Animals were sampled randomly from the farm, based on development stages and ponds (3 batches of tadpoles, 1 batch of 20 small frogs and 2 batches of 8 adults) for pan-pathogen examination. All the selected batches were apparently healthy except for one batch of adults that showed lesions: swollen joint (*n* = 1) and subcutaneous edema (*n* = 2), confirmed at necropsy. After necropsy, bacteriological analyses were performed on 6 pools of individuals (whole animal for early stages [20 g] and internal organs for adults), and on visible lesions. A number of regular, brownish colonies, reaching 2 mm after 48 h, were isolated from the only adult batch showing lesions. Testing using the commercial API20-NE identification system (Biomérieux, France) pointed to *Ochrobactrum anthropi*. MALDI-TOF assay (Bruker Daltonics, France) run on a spot of pure culture overlaid with 1 μL of HCCA matrix indicated *Brucella* spp. using the Biotyper Security-Related (SR) database (26). *Brucella* misidentification using commercial biochemical tests is frequently reported (27); (28), and can result in laboratory-acquired infections (29, 30). Isolates were subsequently sent to the national reference laboratory for reliable identification and refined characterization.

### Phenotypic identification

Standard phenotypic identification (31) confirmed the *Brucella* genus (Table 1), without concluding unequivocally on species determination. Interestingly, strain biotyping traits were not strictly consistent with the *B. inopinata*-like profile previously described in anurans, in particular due to phage lysis. Surprisingly, phenotypic features (Table 1) were closer to the *B. microti* reference strain CCM 4915, except for the A-dominant agglutination pattern, already described for one *B. microti* fox isolate (32).

**Table 1:** Classical phenotypic characterization of the frog isolate investigated in this study *vs. B. inopinata, B. inopinata*-like strains isolated from exotic frogs, and *B. microti* field/reference strains.

	***B. inopinata***	***B. inopinata*-like**	***B. microti***	**17-2122-4144**
Morphology	S	S	S^a^	S
CO_2_	–	–	–	–
H_2_S	+	–^b^	–^c^	–
Oxidase	+	+	+	+
Urease	+ rapid	+^d^	+ slow	+ slow
A	–	–^e^	–^f^	+
M	+ weak	–^e^	+ ^g^	–
R	–	–	–	–
Thionin	+	+	+	+
Fuchsin	+	+	+	+
Tb RTD	–	–	–	–
Tb 10^4^ RTD	+ PL	–	+	+
Wb RTD	ND	–	+	+
Iz RTD	ND	–	+	+
R/C RTD	ND	–	ND	–

*R/S, Colony morphology (Rough/Smooth); CO_2_, CO_2_ requirement; H_2_S, H_2_S production; Agglutination with monospecific A, M and R (rough) antisera; Dye (thionin and basic fuchsin) concentration 20 μg.mL^−1^ in serum dextrose medium (1/50,000); +, Growth or Lysis by phages; –, No growth or no lysis; PL, Partial lysis*.

a *Some rough isolates from soil*.

b *Some isolates positive*.

c *One strain positive*.

d *Various rates*.

e *Some isolates A+ M-*.

f *One fox isolate: A+ M-*.

g *Rough isolates from soil: A+, M+, R+; (31, 32)*.

Growth kinetics in nutritive tryptic soy and M9 minimal broths confirmed faster growth than classical fastidious *Brucella* for the investigated frog strain, named 17-2122-4144, with a generation time identical to *B. microti* CCM 4915 (i.e., approximately 4 h in our growth conditions).

Moreover, antimicrobial susceptibility testing (AST) performed by thedisk and E-test methods highlighted an identical pattern of susceptibility to the main anti-*Brucella* antibiotics of veterinary and human interest: doxycycline (DX), rifampicin (RIF), streptomycin (STM), ofloxacin (OFX), and sulfamethoxazole/trimethoprim (SMX/TMP) for the strain 17-2122-4144 *vs B. microti* CCM 4915.

### Molecular analysis

Conventional genus- and species-specific PCR methods (33) were performed (Table 2). The real-time PCR assays confirmed that the investigated strain belongs to the *Brucella* genus. The obtained Bruce-Ladder pattern was shared with the *B. microti* and *B. suis* biovar 2 reference strains and was distinct from other *Brucella* reference and vaccine strains. The Suis-Ladder method split the biovars of *B. suis, B. canis* and *B. microti* as previously described (32), and concluded that there was a single pattern between *B. microti* and the investigated strain.

**Table 2:** Molecular characterization of the frog isolate investigated in this study vs. *B. inopinata, B. inopinata*-like strains isolated from exotic frogs, and *B. microti* field/reference strains.

	***B. inopinata***	***B. inopinata*-like**	***B. microti***	**17-2122-4144**
RT-PCR	+	+	+	+
Bruce-Ladder	NR	NR	*Bmic*/*Bsuis* bv2	*Bmic*/*Bsuis* bv2
Suis-Ladder	NR	NR	*B. microti*	*B. microti*
RFLP	NR	NR	*B. microti*	Different from *B. microti*
AMOS-ERY	NR	NR	*B. microti*	Close to *B. suis*
MLVA16	*B. inopinata*	NR	*B. microti*	*B. microti*
ANI^*^ (%)	98.33	97.77–98.2	99.89	100
11.7 kbp insertion	–	–	+	+
Ectoine system	–	+	–	–
16S rRNA (5 mutations)	+	+	–	–
*recA* (*B. microti*-specific SNP)	–	–	+	+
LPS pattern	*B. inopinata*	Close to *B. inopinata*	*B. microti*	*B. microti*
MLST-21	*B. inopinata*	Close to *B. inopinata*	*B. microti*	Close to *B. microti*
HRM PCR	NR	NR	*B. microti*	*B. microti*
wgSNP	*B. inopinata*	*B. inopinata*-like	*B. microti*	*B. microti*

* *ANI values are calculated on the basis of the reference strain vs. the frog strain investigated in this study; NR, not reported; Bmic/Bsuis bv2, the pattern is shared with B. microti and B. suis bv 2 reference strains; +, presence; –, absence; B. microti, the pattern is a unique signature among B. microti strains described to date; B. inopinata, the pattern is a unique signature among B. inopinata strains described to date; B. inopinata-like, the pattern is a unique signature among B. inopinata-like strains described to date (25)*.

Although most conventional molecular techniques did not make it possible to differentiate between CCM 4915 and 17-2122-4144, minor differences were observed regarding RFLP results (34): the restriction profile of the *omp*2b target digested by *Eco*RI for 17-2122-4144 was distinct from the CCM 4915 profile, but similar to the *B. pinnipedialis* reference strain B2/94. Interestingly, the AMOS-ERY profile of the studied strain (2 fragments of 1.3 kbp and 1.2 kbp) was divergent from classical *Brucella* spp. profiles, as well as, from the atypical *B. microti* (one single 1.3 kbp fragment), but close to *B. suis* reference strains (1.3 and 1.2 kbp).

**Figure 1:**
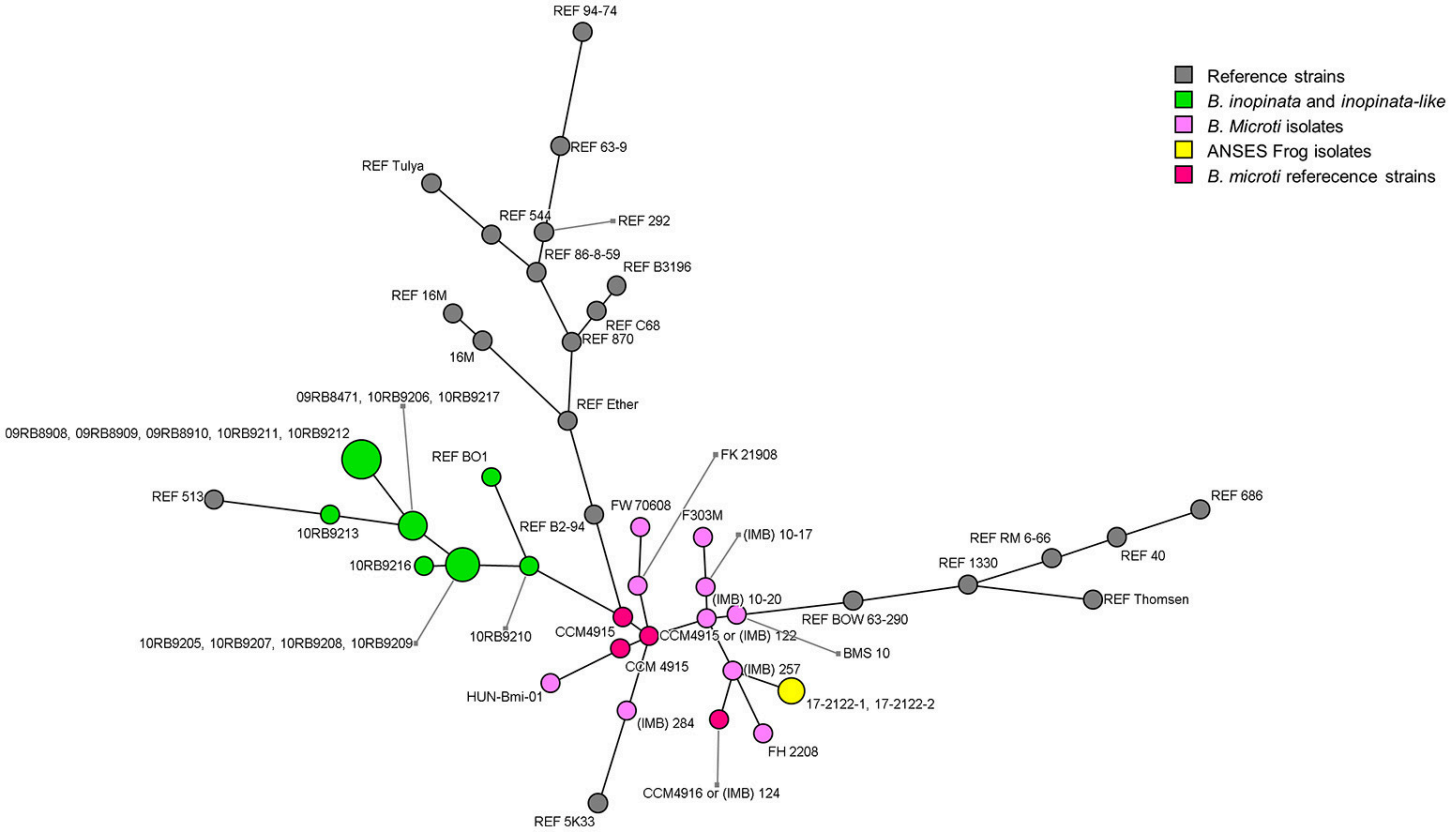
Minimum Spanning Tree of MLVA-16 genotypes of the frog strain investigated in this study, *B. microti* isolates published to date, and all *Brucella* reference strains. *B. microti* isolates are distinguished by different colors: yellow for the frog strain investigated in this study; pink for previously published isolates (32); red for *B. microti* reference strains; other reference strains are colored in gray.

In addition to classical molecular approaches, phylogenomic methods were used (Table 2). Unsurprisingly, MLVA-16 results showed that 17-2122-4144 clustered within *B. microti* reference strains CCM 4915 and CCM 4916 and together with the 10 field strains reported to date (Figure 1; Supplementary Figure 1), close to the *B. neotomae* reference strain 5K33 (32).

*De novo* assembly showed a genome with a total length of 3,335,258 bp, vs 3.37 Mbp for *B. inopinata* BO1 and 3.34 Mbp for *B. microti* CCM 4915. Moreover, the total number of predicted genes per genome (evaluated by QUAST) for 17-2122-4144 (3,141 genes) is very similar to CCM 4915 (3,145 genes), closer than for BO1 (3,220 genes). ANI exhibited maximum identity with *B. microti* CCM 4915 (99.89%); 98.33% identity with *B. inopinata* and 97.77–98.2% with 3 frog *Brucella* genomes from the NCBI database (24). Similarly, a bacteriophage-related 11,742 bp insertion, previously described as present only in *B. microti* isolates (15), was also found within the investigated genome. Further analyses using Roary and Scoary to compare gene presence or absence did not underline any gene signature specific to the investigated field isolate vs. *B. inopinata* BO1, *B. microti* CCM 4915 and *B. melitensis* bv1 16M. Moreover, the ectoine system, conferring salt and temperature resistance, described in atypical *Brucella* (24), was absent in 17-2122-4144, as well as, BO1 and CCM 4915. Similarly, 16S rRNA and *recA* comparative analyses (27) confirmed that 17-2122-4144 was closely related to *B. microti*, with absence of 5 *rrs* mutations in 17-2122-4144 and CCM 4915, systematically present in *B. inopinata* and *B. inopinata*-like strains, and presence of a single *recA B. microti*-specific SNP in 17-2122-4144 (24, 25, 32).

In line with previous studies (4, 5, 25, 35), we assessed *in silico* the LPS profile of the investigated isolate, especially focusing on the genes essential for LPS synthesis: the *wbk* region, *wboA* and *wboB* genes, the *manBCA* region, as well as, the *tagH* and *rfbD* genes. Regions of the investigated isolate were strikingly similar to *B. microti*. In addition, our analysis concluded presence of the *wboA, wboB* and *manBCA* genes (unlike bullfrog strains, BO2 and B13-0095) and absence of the *rmlACBD* region and *tagH* gene found in BO2 and B13-0095 in the investigated genome. Interestingly, the *rfbD* gene was present in 17-2122-4144, but disrupted by numerous stop codons, as in *B. microti* CCM 4915. Our results show that the LPS profile of the novel isolate matches that described in *B. microti*.

*In silico* MultiLocus Sequence Typing-21 (MLST-21) confirmed this genetic proximity of 17-2122-4144 with *B. microti* CCM 4915. Except for the *mutL* gene involved in DNA mismatch repair, which harbored a point mutation at position 1149 (E383V), the MLST-21 pattern was strictly identical between the novel frog isolate and *B. microti*.

**Figure 2:**
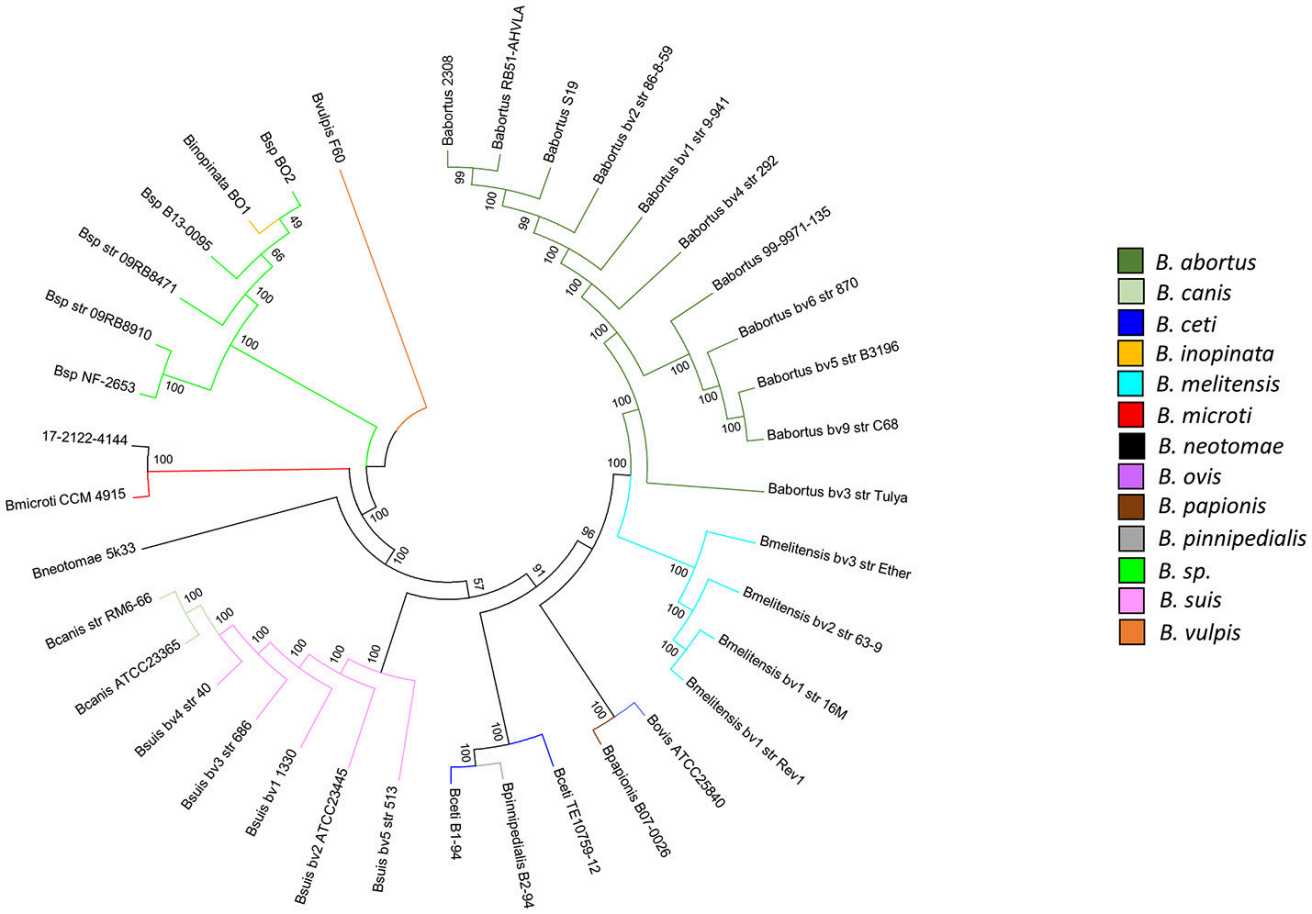
Phylogenetic comparative whole-genome SNP analysis of the frog strain investigated in this study and all *Brucella* reference strains. The dendrogram was constructed using the maximum likelihood method with 200 bootstrap repetitions (36,590 SNPs). Species are distinguished by different colors. A log scale is used in this tree, allowing a better distinction between isolates.

In parallel, *B. microti* and *B. inopinata-*specific High Resolution Melting (HRM) PCR assays were designed and performed against 17-2122-4144, emphasizing a profile similar to *B. microti* and divergent from *B. inopinata*. Phylogenetic comparative whole-genome SNP analysis showed that 17-2122-4144 is very close to *B. microti* CCM 4915 (323 SNPs without filtering, 73 SNPs in an overall phylogeny context) among the classical *Brucella* group (Figure 2; Supplementary Table 2), unlike strains previously isolated from frogs that clustered with *B. inopinata* in the “early-diverging” *Brucella* group (25).

### Taxonomic conclusions

The investigated frog strain is very close to *B. microti* species, and not to *B. inopinata*-like strains, as might be expected given the current taxonomy of strains isolated from frogs. Despite minor polyphasic discrepancies, 17-2122-4144 is qualified as a *B. microti*-like strain.

*B. microti* has been isolated from wild animals, such as the common vole *Microtus arvalis* (36), (7), wild boars (37), and red foxes (9), and is described as persistent over a long period in soil (8), suggesting the existence of environmental reservoirs. Interestingly, although *B. microti* is suspected to induce epizootic mortality in the common vole (36), isolated cases from other described hosts seem to be asymptomatic, with no associated clinical signs (9, 37), suggesting asymptomatic carriage. In addition, the replication ability of *B. microti* was demonstrated in mouse macrophages (25, 38) and its pathogenic potential was shown to cause death in murine models (38–40) and lesions in chicken embryo models (41).

Anthropogenic interference has previously been reported to impact brucellosis prevalence in wildlife (42), raising questions on the influence of natural selection and selective breeding on *B. microti* fitness. Long-term environmental persistence outside the host and the putative ubiquitous nature of the *B. microti*-like strain investigated in this study, as well as, its unknown—but suspected—animal pathogenic and zoonotic potential, raise possible concerns for animal and public health. Further epidemiological investigations in wild frogs, as well as, in the natural environment might be required to offer new insights regarding bacterial carriage and possible clinical expression, depending on housing conditions. Moreover, *in vitro* cell infection experiments, as well as, *in vivo* infections will be required to determine the pathogenic potential of the *B. microti*-like isolates from frogs, in accordance with previous approaches applied to amphibian strains (25).

This study is the first isolation of *B. microti*-like bacteria from *P. ridibundus* on a domestic frog farm in France.

## Materials and methods

### Bacterial strains and genomes

Strains and/or genomes used in this study are listed in Supplementary Table 1.

### Phenotypic identification

Isolates were characterized using standard procedures (31) in BSL-3 facilities. AST was performed by the disk (Thermo Scientific - Oxoid) and E-test (Biomerieux) diffusion methods on Mueller-Hinton agar plates, supplemented with 5% sheep blood (DX, RIF, STM, OFX, SMX/TMP), following the recommendations of the Clinical and Laboratory Standards Institute (43). Growth kinetics were performed in nutritive tryptic soy and on M9 minimal broths (44). Stationary phase cultures were diluted to an OD_600_ of 0.03 and grown in 75 cm^2^ cell culture flasks at 37°C. OD_600_ was measured every hour, and each point was serially diluted and plated on *Brucella* agar to determine colony-forming units. Each strain was assayed in triplicate.

### Molecular analysis

Genomic DNA was extracted using the High Pure PCR template preparation kit (Roche Diagnostics, France), according to the manufacturer's instructions.

Real-Time PCR (45), Bruce-Ladder (46), Suis-Ladder (47), RFLP-PCR (34), AMOS-ERY (48) and MLVA-16 (49) assays were performed as previously described. All tests have been carried out in duplicate (i.e., from 2 independent isolates). Clustering analysis was performed by using a minimum spanning tree (MST) and the cophenetic correlation coefficient with the UPGMA algorithm from MLVA data (Bionumerics v7.6.2; Applied Maths, Belgium), as well as, a maximum likelihood tree based on the Jukes Cantor model (with 200 repetitions for bootstrap) from WGS data (Bionumerics 7.6.2 and MEGA software v. 6).

Whole-genome sequencing (Illumina HiSeq2500 platform, 100X) was performed. *De novo* assembly was performed using the SPAdes v3.9 algorithm. QUAST 4.6.3 was used to assess assembly robustness by gathering extensive assembly statistics. Nucleotide sequences of contigs from this work were deposited in the European Nucleotide Archive (EMBL-EBI) –Bioproject: http://www.ebi.ac.uk/ena/data/view/PRJEB26927; Accession Number: ERZ654921–. Average Nucleotide Identity (ANI) values were calculated using Jspecies (50). Phylogenetic SNP distances were determined using the Bionumerics v7.6.2 wgSNP-module. Roary v3.6.1 and Scoary were used to generate and compare matrices of gene presence/absence. Polymorphism of 16S rRNA (27), *recA* (27), the ectoine system (24), the LPS pattern (4, 5, 25, 35) as well as, the presence of a bacteriophage-related 11,742 bp insertion (15) were studied as previously described, using Bionumerics v7.6.2 for multiple sequence alignments. The 21 locus scheme (MLST-21) was determined *in silico* as previously described (33, 51).

HRM PCR assays were carried out as previously described (52) using the Bmic_1F (5′-AACTGCCGGATGTGAAAAAG-3′) and Bmic_1R (5′-AAGGATCGAGGCGTCATAAA-3′) primers.

## Author contributions

MJ, GG, and VM designed the study and wrote the paper. FR, P-HP, and EP carried out preliminary identification studies. MJ and BT performed standard bacteriology. LP and GG performed growth kinetics and antimicrobial susceptibility tests. LP, BT, TV, and GG contributed to molecular studies. GG and VM performed bioinformatics analyses. GG, MJ, CP, and VM performed data interpretation. All authors read and approved the manuscript content.

### Conflict of interest statement

The authors declare that the research was conducted in the absence of any commercial or financial relationships that could be construed as a potential conflict of interest.

## Supplementary material

Supplementary Figure 1 MLVA-16 analysis of the frog strain investigated in this study, *B. microti* isolates published to date and all *Brucella* reference strains. The dendrogram was constructed with the cophenetic correlation coefficient and UPGMA algorithm. *B. microti* isolates are distinguished by different colors: yellow for the frog strain investigated in this study; pink for previously published isolates (32); red for *B. microti* reference strains; other reference strains are colored in gray.

Supplementary Table 1 *Brucella* strains used in this study.

Supplementary Table 2 List of identified SNPs among the novel frog isolate investigated in this study and *Brucella* strains, as well as, location of mutations found within 17-2122-4144 vs. CCM4915.
