# Supplementary figures and images for: Phenotypic and Molecular Characterization of Brucella microti-Like Bacteria From a Domestic Marsh Frog (Pelophylax ridibundus)
Source: Front Vet Sci. 2018 Nov 15;5:283. doi: 10.3389/fvets.2018.00283 (PMC6249338; doi:10.3389/fvets.2018.00283)

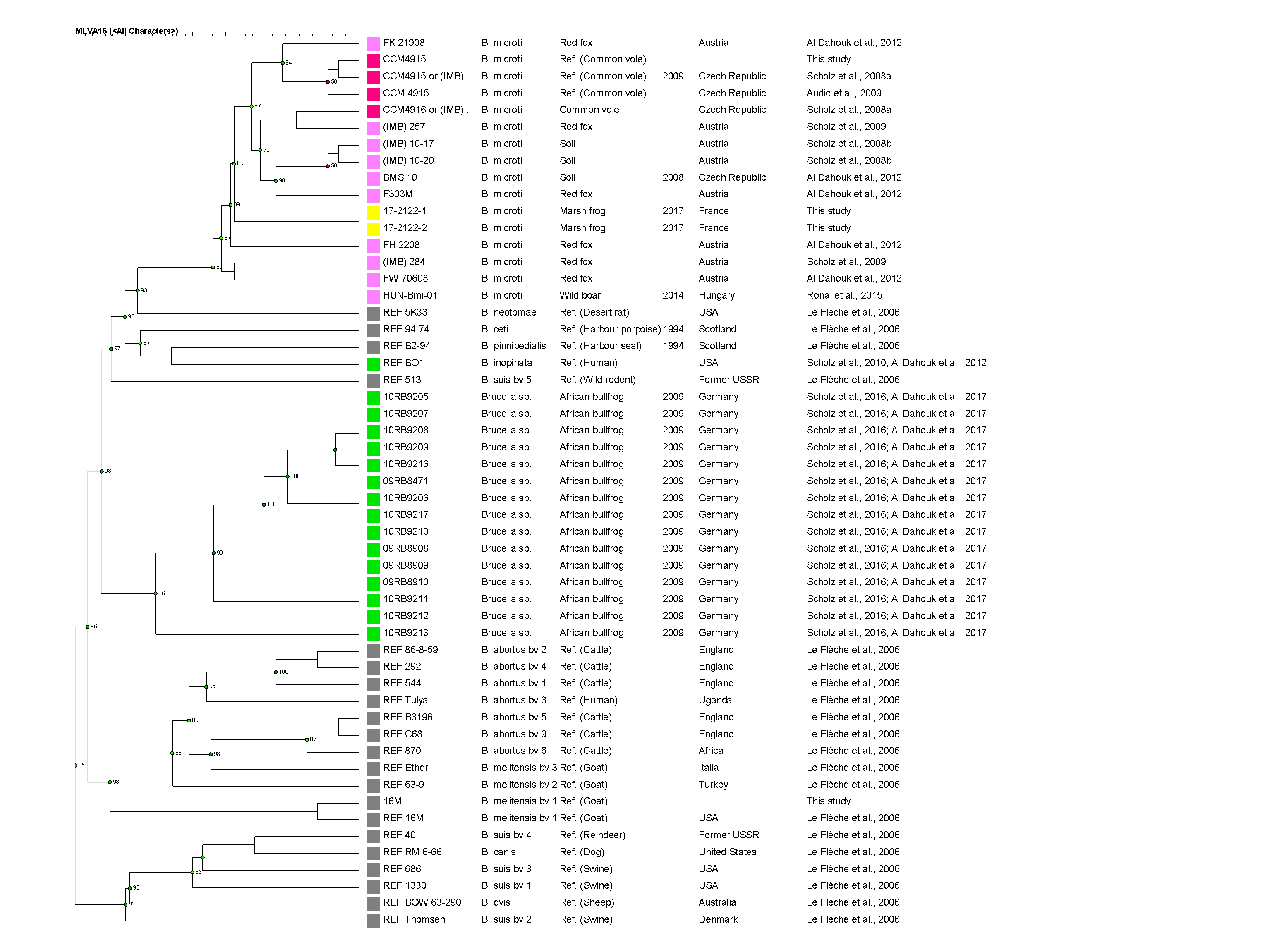

Supplement: Supplementary Figure 1 — MLVA-16 analysis of the frog strain investigated in this study, B. microti isolates published to date and all Brucella reference strains. The dendrogram was constructed with the cophenetic correlation coefficient and UPGMA algorithm. B. microti isolates are distinguished by different colors: yellow for the frog strain investigated in this study; pink for previously published isolates (32); red for B. microti reference strains; other reference strains are colored in gray. [file Image_1.TIF]
